# Microglia in Post‐Traumatic Brain Injury (TBI) Cognitive Impairment: From Pathological Changes to Therapeutic Approaches

**DOI:** 10.1111/cns.70568

**Published:** 2025-08-18

**Authors:** Ningcen Li, Wenhui Lu, Limei Tang, Lina Zhu, Weibin Deng, Hang Liu, Changquan Huang, Jingying Jin, Jingjiao Zeng, Shitai Chen, Lianqi Geng, Xiuwu Hu, Liang Zhou

**Affiliations:** ^1^ Research Center of Experimental Acupuncture Science Tianjin University of Traditional Chinese Medicine Tianjin China; ^2^ Affiliated Rehailitation Hospital of Nanchang University Jiangxi China; ^3^ Acupuncture and Moxibustion Department Nanchang Hongdu Hospital of Traditional Chinese Medicine Jiangxi China; ^4^ Acupuncture and Moxibustion Medical Clinical Research Center of Jiangxi Province Jiangxi China; ^5^ Fourth Teaching Hospital of Tianjin University of TCM Binhai New Area Hospital of TCM Tianjin Tianjin China

**Keywords:** dynamic and dual roles, microglia, neuroinflammation, pathological changes, post‐TBI cognitive impairment, therapeutic approaches, traumatic brain injury

## Abstract

**Background:**

Traumatic brain injury (TBI), as a common and serious neurological disease, brings enormous physical and psychological burden to patients. Among them, cognitive impairment caused by TBI greatly affects the quality of life and social function of patients. Microglia, as key immune cells in the central nervous system, play a crucial role in the occurrence and development of cognitive impairment after TBI. This review delves into the important functions of microglia in normal physiological states and their multifaceted manifestations in post‐TBI cognitive impairment.

**Method:**

A systematic literature review was conducted using PubMed, Google Scholar, Web of Science and Scopus, with a focus on preclinical studies as well as clinical trials published in the past 20 years. The key search terms include “traumatic brain injury,” “cognitive impairment,” “microglia,” etc.

**Results:**

During the acute phase of TBI injury, microglia rapidly activate, clear injury debris, and initiate repair, reducing secondary injury. At the same time, microglia undergo phenotype polarization during this stage. Some M1‐type microglia can release various inflammatory factors through inflammation‐related pathways, triggering inflammatory signals and leading to neuronal apoptosis and neuroinflammatory responses. M1 polarization driven persistent inflammation becomes an important factor in the chronic progression of TBI, leading to cognitive impairment. On the other hand, the phagocytic function of activated microglia also changes, which may lead to excessive phagocytosis of normal neurons and synapses, causing synaptic dysfunction and further exacerbating cognitive impairment. Meanwhile, insufficient clearance of damaged cells and debris can lead to persistent inflammation, hindering nerve repair. This review also provides a detailed introduction to potential treatment methods. This includes inhibiting the activation of microglia and the release of inflammatory factors through anti‐inflammatory therapy, regulating the phenotype of microglia to promote their transformation to M2 type, promoting the normalization of microglial phagocytic function, regulating the structure and function of synapses, and using stem cell therapy to secrete neurotrophic factors to regulate microglial function. The strategy of integrating traditional Chinese and Western medicine is also a good direction.

**Conclusions:**

Microglia are both the “driving force” of neuroinflammation and the “key executor” of repair in post‐TBI cognitive impairment. Their dual effect is dynamically influenced by multiple factors. Future treatments require precise targeting of polarization balance, combined with spatiotemporal specific intervention strategies, to break the vicious cycle of chronic inflammation and promote neurological function recovery.

## Graphical abstract

This review systematically analyzes the multi‐stage dynamic pathological evolution of microglia and therapy strategies targeting microglia in PSCI, providing a new perspective for a deeper understanding of the microscopic mechanisms of the disease.

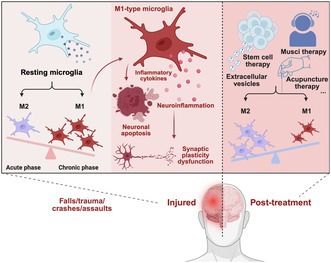

**Abbreviations**
ad
: alzheimer's disease
AIF: apoptosis‐inducing factor
Apaf‐1: apoptosis protease activating factor‐1
BBB: blood–brain barrier
CCI: controlled cortical impact injury
CCL2: chemokine C‐C motif ligand 2
CHAT: choline acetyltransferase
CNS: central nervous system
DAMPs: damage associated molecular patterns
DG: dentate gyrus
DISC: death inducing signaling complex
DP: 3,6′‐dithiopomalidomide
EP: ethyl pyruvate
FADD: fas associated death domain protein
GAD2: glutamate decarboxylase 2
GDNF: glial cell‐derived neurotrophic factor
GSDMD: gasdermin D
HMGB1: high mobility group box 1
hNSCs: human fetal neural stem cells
hUC‐MSCs: human umbilical cord‐derived mesenchymal stem cells
IMiD: immunomodulatory imide drug
Itpkb: inhibits 1,4,5‐trisphosphate 3‐kinase B
LRRK2: leucine‐rich repeat kinase 2
LTP: long‐term potentiation
MAP 2: microtubule‐associated protein
MBIs: music based interventions.
MMP‐9: matrix metalloproteinase‐9
NAD: nicotinamide adenine dinucleotide
Nlg1: neuroligin1
NLRP3: NOD‐pyrin‐domain‐containing protein 3
OBB: oxyberberine
PACAP: pituitary adenylate cyclase‐activating polypeptide
PAR‐2: proteinase‐activated receptor‐2
PARP: poly ADP ribose polymerase
RAGE: receptor for advanced glycation end product
RNS: reactive nitrogen species
ROS: reactive oxygen species
Syn1: synapsin‐1
Syt1: synaptotagmin1
TBI: traumatic brain injury
TRADD: TNF receptor associated death domain protein
TRAF: TNF receptor‐associated factor
VEGFR2: vascular endothelial growth factor receptor 2
VGlut2: vesicular glutamate transporters 2
VIP: vasoactive intestinal peptide
ZO‐1: zonula occludens‐1

## 1 Background

With the rapid development of transportation and construction worldwide, accidents have occurred frequently, resulting in brain damage that is the main cause of patient death and disability. The latest research on traumatic brain injury (TBI) indicates that it remains a significant global public health concern. Globally, in 2019, TBI had 27.16 million new cases, 48.99 million prevalent cases, and 7.08 million years lived with disability [1]. The burden of TBI is particularly high in low‐ and middle‐income countries, where healthcare systems often lack the resources for optimal care [2]. In the past, TBI was defined as an injury event with limited recovery, and now it is also considered a chronic disease that can affect multiple functional areas, some of which may worsen over time. A study has found that many people with TBI are still in a state of moderate to severe disability at 5 years [3]. As a common neurological injury, TBI can cause various symptoms, ranging from simple headaches to permanent memory and thinking problems. Head injuries can be divided into primary injuries and secondary injuries. The former occurs immediately after trauma, while the latter includes a series of injuries that may occur several hours or even days later [4]. After TBI, cognitive impairment is a common and significant sequela that affects patients' daily life and social function. The main cognitive impairments include memory impairment, lack of concentration, executive dysfunction, and slowed information processing speed. The cortex and hippocampus are the two brain structures most susceptible to significant effects in brain injury [5]. Due to the important role of hippocampal neurons in learning and memory function, their damage may lead to a risk of memory dysfunction [6].

Glial cells, especially microglia, play a crucial role in the homeostasis and diseases of the central nervous system (CNS). Evidence suggests that glial cells are influenced by TBI [7]. A study evaluated whether TBI leads to spatiotemporal changes in the phenotype of microglia and whether it affects the sensory, motor, or learning and memory functions of TBI mice. It was found that TBI results in a spatiotemporal increase in activated microglia at 35 days post‐TBI, which was negatively correlated with spatial learning and memory [8]. Thoroughly summarizing the mechanism of cognitive impairment mediated by post‐TBI glial cells is of great significance for determining the therapeutic window, accelerating translational research, and improving patient prognosis.

## 2 Microglia (MG) in Post‐TBI Cognitive Impairment

The name microglia was first (1919) proposed by Pío del Río Hortega (1882–1945), accounting for approximately 5% to 15% of all brain cells [9]. It is believed to be a phagocytic cell responsible for clearing debris during CNS development and disease processes. After a century of development, the roles of microglia in immune surveillance, synaptic remodeling, and nourishing nerves have gradually been explored [10]. Nowadays, microglia are considered innate immune cells that reside in the CNS, resembling macrophages and possessing memory‐like functions [11]. As a multifunctional cell, it can interact with many other cells in the CNS, including neurons, astrocytes, and oligodendrocytes, etc. [12]. With the development of various experimental techniques, researchers have found that microglia also play an important decisive role in the occurrence and development of CNS diseases [13], such as post‐TBI cognitive impairment. Phosphorylated microtubule‐associated protein tau aggregates are pathological markers of various neurodegenerative diseases. A study has found that on the first day after TBI, the expression of leucine‐rich repeat kinase 2 (LRRK2, a kinase associated with p‐tau) in microglia at the site of cortical injury is upregulated and continues until the 10th day [14]. Microglia may also become a new promising therapeutic target for post‐TBI cognitive impairment.

### 2.1 Neuronal Apoptosis

The hippocampus is an important area for memory formation and consolidation, and neurons in the hippocampus are crucial for memory formation and maintenance [15]. In the experimental injury model of BI, although the main damage is to the cortex below the impact site, it is interesting to note that the hippocampus undergoes neuropathological changes and is subjected to more substantial functional damage [16]. After TBI, neuronal death is one of the main primary injuries. Neurons, as one of the most long‐lived cells, have two main forms of death: apoptosis and necrosis [17]. Cognitive impairment is closely related to neuronal apoptosis and death. Neuronal necrosis is a non‐programmed cell death, a passive process characterized by cell membrane rupture, triggering an inflammatory response [18]. Neuronal apoptosis is a programmed cell death process that is actively controlled by genes. Apoptosis generally does not cause inflammatory reactions, but in some cases, if apoptotic cells are not cleared in a timely manner, leakage of contents may cause local inflammatory reactions. Meanwhile, the apoptotic bodies involved in the process of apoptosis are not immediately engulfed and may be recognized by immune cells, leading to inflammation [19]. The main initiating molecules of the apoptosis program include apoptosis protease activating factor‐1 (Apaf‐1), Bcl‐2 family, and caspase family proteins [20]. Here, we mainly introduce microglia‐mediated neuronal apoptosis in post‐TBI cognitive impairment, as well as acute and secondary apoptosis that exacerbate neuronal damage and functional loss after TBI.

Apoptosis leads to the loss of neurons in the hippocampus, directly affecting memory function. After TBI, microglia are considered the first line of defense in the brain and quickly activate within a few minutes, then proliferate and migrate to the site of injury [21]. Microglia play an important role in neuronal apoptosis. The intrinsic and extrinsic pathways of neuronal apoptosis are mediated by microglia involving different initiating molecules and signaling mechanisms [19]. The intrinsic pathway is triggered by signals from within the cell, usually in response to internal stress such as oxidative stress, DNA damage, Ca^2+^ overload, and hypoxia. (1) Microglia, when activated, produce a large amount of reactive oxygen species (ROS) and reactive nitrogen species (RNS), such as superoxide, hydrogen peroxide, nitric oxide, etc. These ROS can enter neurons, increase pro‐apoptotic proteins (e.g., Bax, Bak) which can promote cytochrome c release, and anti‐apoptotic proteins (e.g., Bcl‐2, Bcl‐xL) which can inhibit the activity of Bax and Bak and maintain mitochondrial membrane integrity, further causing mitochondrial damage, resulting in loss of mitochondrial membrane potential and neuronal apoptosis [22]. (2) TBI triggers an inflammatory response, where microglia are activated and migrate to the site of injury, releasing various inflammatory factors and inducing neuronal apoptosis by activating downstream signaling pathways, such as caspase‐3. TBI can increase the activated microglia and inhibit the θ, β, and γ oscillation power in hippocampal CA1, then exacerbate cholinergic, glutamatergic, and GABAergic neuronal damage by decreasing microtubule‐associated protein 2 (MAP2) and neurotransmitter‐associated proteins such as glutamate decarboxylase 2 (GAD2), vesicular glutamate transporters 2 (VGlut2), choline acetyltransferase (CHAT) intensity, and increasing caspase‐3, leading to the reduction of recognition ability [23]. (3) The glutamate released by microglia can overactivate the N‐methyl‐D‐aspartate receptor (NMDAR) of neurons, leading to excessive Ca^2+^ influx. Ca^2+^ enters mitochondria, causing mitochondrial dysfunction and releasing pro‐apoptotic factors such as cytochrome c. Recently, some studies have also emphasized the crucial role of glutamatergic transmission‐mediated changes in microglia–neuron dialogue in cognitive function [24, 25]. The activation of extrinsic pathways is achieved through the binding of ligands to death receptors [26]. (1) Activated microglia can express and release death ligands, such as Fas ligand (FasL) and TNF‐α. These death ligands bind to death receptors on neurons, such as Fas and TNF receptors, forming a death‐inducing signaling complex (DISC) with Fas‐associated death domain protein (FADD), activating caspase‐8 and further activating downstream effector caspase‐3, initiating extrinsic apoptotic pathways. In unilateral cortical impact injury‐induced experimental TBI rats, it was found that from 15 min to 72 h after trauma, the expression of Fas and FasL increased in microglia in the ipsilateral cortex of the injury site, while Fas and FasL were not expressed in the contralateral cortex and hippocampus. However, after 14 days, there was no evidence of Fas and FasL immune‐positive cells in the hippocampus on the same side of the injury site. This suggests that Fas and FasL may be involved in the pathological and physiological mechanisms of early apoptotic neurodegeneration after TBI [27]. Besides, inflammatory factors released by microglia, such as TNF‐α, can recruit TNF receptor‐associated death domain protein (TRADD) and TNF receptor‐associated factor (TRAF) through the TNF receptor, activate caspase‐8, and initiate the extrinsic apoptotic pathway [28, 29].

**FIGURE 1:**
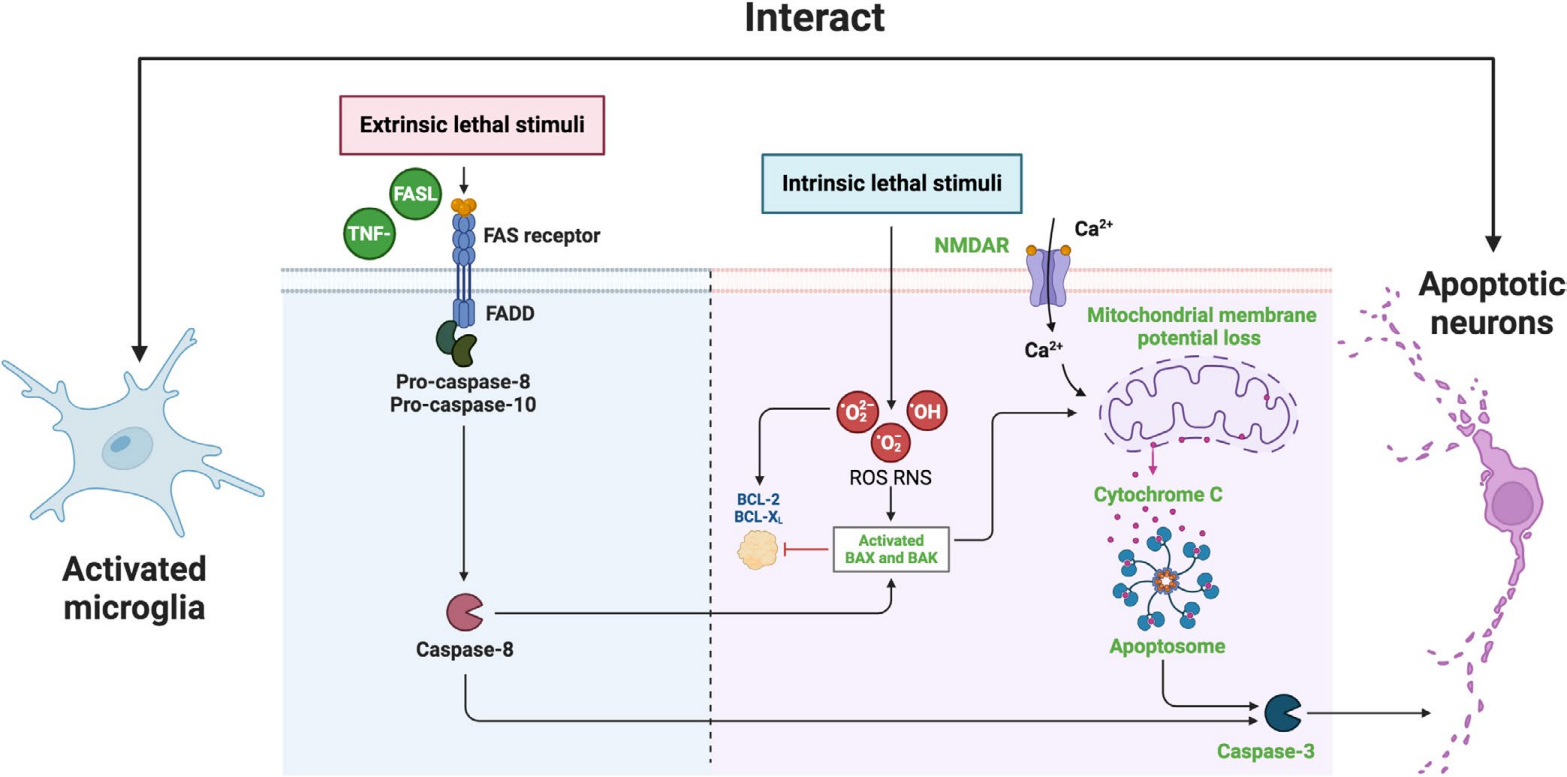
Microglia mediated neuronal apoptosis in post‐TBI cognitive impairment. The intrinsic pathway is triggered by signals from within the cell, usually in response to internal stress such as oxidative stress, DNA damage, Ca^2+^ overload and hypoxia. The activation of extrinsic pathways is achieved through the binding of ligands to death receptors. FADD, fas associated death domain protein; FasL, fas ligand; NMDAR, N‐methyl‐D‐aspartate receptor; RNS, reactive nitrogen species; ROS, reactive oxygen species.

In addition, apoptosis can also lead to a chronic inflammatory response, further damaging neurons and affecting cognitive function. Neuron apoptosis can also lead to the loss of connections between neurons and synapses, affecting synaptic plasticity and decreasing learning ability. The death of neurons in the cortex and hippocampus can also disrupt neural networks, affecting the processing and transmission of information in different brain regions, further leading to the destruction of attention, executive function, and spatial reasoning ability (Figure 1).

### 2.2 Neuroinflammation

The pathology of post‐TBI cognitive impairment is complex, mainly caused by secondary injury [30]. After primary mechanical injury, brain tissue is mechanically damaged, leading to disruption of the blood–brain barrier (BBB), release of cell debris and various damage‐associated molecular patterns (DAMPs) that can activate microglia [31]. As a key innate immune defense required to protect the brain from damage, after TBI, activated microglia will change their morphology, transitioning from a resting state to an activated state; resident microglia are rapidly activated and transferred to the damaged area, which can clear debris but can also cause an inflammatory cascade [32]. In response to harmful stimuli, microglia can be activated into the classical M1 phenotype to release pro‐inflammatory cytokines, or activated into the alternative M2 phenotype to release anti‐inflammatory cytokines, resolve inflammation, clear damage, and repair tissues [33]. Research has shown that sustained and excessive neuroinflammatory responses mediated by microglia after TBI can lead to trauma‐induced neurodegeneration, mainly manifested as cognitive impairment [34]. It is reported that dysregulated microglial activation can persist for up to 17 years in TBI patients [35]. On the one hand, the characteristic of neuroinflammation is the release of pro‐inflammatory cytokines and activation of innate immune responses by immune cells in the CNS. Activated microglia produce a large amount of ROS and RNS, which can cause oxidative damage to cells and tissues. Oxidative stress not only directly damages neurons and other glial cells but also induces activated M1‐type microglia to release various pro‐inflammatory cytokines, such as TNF‐α, IL‐1β and IL‐6, by activating inflammatory signaling pathways such as NF‐κB. These cytokines further amplify the inflammatory response, leading to more neuronal damage and cell death [36]. High mobility group box 1 (HMGB1) protein is considered to be a member of the DAMP family [37]. Once released into the extracellular space from necrotic or activated cells, it can play a key role in triggering inflammatory responses in TBI through the activation of multiple receptors such as the receptor for advanced glycation end product (RAGE) and toll‐like receptor in microglia [38, 39, 40]. Inflammasome‐mediated signaling has been identified as playing important roles in the M1‐type microglia activation after TBI. Among the family of inflammasomes, NOD‐, LRR‐, and pyrin‐domain‐containing protein 3 (NLRP3) have become major research targets due to their abundant expression in microglia [41]. Research has found that at 24 h after TBI, the expression of products of the cGAS STING signaling pathway, such as phosphorylated TBK1, IRF3, and IFN‐β mRNA, increased. The activation of STING can further induce activation of the NLRP3‐dependent inflammasome, exacerbating pyroptosis‐related neuroinflammation that contributes to significant emotional impairment through mediating hippocampal injury. At 32 days post‐TBI, activation of M1‐type microglia was further investigated in the hippocampus, which may be caused by inflammation related to pyroptosis [42]. Some studies have shown that TBI is also related to the occurrence of neurodegenerative diseases such as Alzheimer's disease (AD). For example, the incidence rate of AD is related to the severity of TBI, and people who have experienced TBI also suffer from AD earlier than people who have not experienced TBI [43, 44]. There is experimental evidence to suggest that TBI promotes neuroinflammation mediated by microglia in the hippocampus of APP/PS1 mice, as well as the transformation of microglia into M1 pro‐inflammatory phenotypes, accelerating cognitive impairment and AD‐like pathology such as Aβdeposition in the APP/PS1 mouse model [45].

MicroRNAs (miRNAs) are a type of noncoding single‐stranded RNA with a length of approximately 19–25 nucleotides. They can degrade mRNA or inhibit target gene translation through specific interactions with target genes after transcription, and can also upregulate target gene translation and transcription levels under specific conditions. Approximately 30% of human genes are regulated by miRNAs, involved in metabolism, growth, and other processes [46]. According to reports, the abnormal expression of various miRNAs in microglia participating in the inflammatory response is associated with the development of TBI. For example, in TBI mice induced by the controlled cortical impact injury (CCI), the expression of miR‐193a was significantly upregulated in ipsilateral cortical tissue and microglia/macrophages isolated from ipsilateral cortical tissue, while the expression of pro‐inflammatory cytokines was significantly enhanced [47]. Some long intergenic non‐protein coding RNA such as LINC00707 that can target miR‐30a‐5p has also been proved increased in the rat cerebral cortex [48].

Chemokines are a type of small molecule protein that can bind to receptors on microglia and activate intracellular signaling pathways, release inflammatory mediators, mediate inflammatory responses, and further induce cognitive impairment after TBI [49]. CX3CL1 and its receptor CX3CR1 are a representative pair of molecules, and a study has found that during the chronic period of 30 days post‐TBI, mice deficient in CX3CR1 that are subjected to CCI exhibit more obvious cognitive impairment and more neuronal death, which may be related to the polarization of M1 phenotype microglia [50].

On the other hand, a sustained inflammatory response can exacerbate the disruption of the BBB, allowing more peripheral immune cells (such as monocytes and macrophages) to penetrate brain tissue, further exacerbating inflammation and tissue damage. Obesity will increase the incidence rate and mortality of cognitive impairment after TBI [51]. The interaction between a high‐fat diet and TBI promotes the transition to chronic reactive microglial/macrophage transcriptome features, as well as related pro‐inflammatory disease states, which form the basis of cognitive impairment. Therefore, targeting the immune cell population may help alleviate post‐traumatic neurodegeneration and neurological dysfunction [52]. A study found that 24 and 72 h after TBI, the expression of tight junction proteins claudin 5 and Zonula occludens‐1 (ZO‐1) was deranged, indicating increased BBB permeability. At the same time, it was found that mast cells were significantly activated and recruited into damaged brain tissues, accompanied by the expression of serum chemokine C‐C motif ligand 2 (CCL2), proteinase‐activated receptor‐2 (PAR‐2), mast cell and inflammation‐associated protein vascular endothelial growth factor receptor 2 (VEGFR2) increased in the brain, and cognitive function of TBI mice is impaired [53]. In addition, inflammatory reactions can also inhibit the proliferation and differentiation of neural stem cells, hinder nerve repair and regeneration, and further exacerbate cognitive impairment.

**FIGURE 2:**
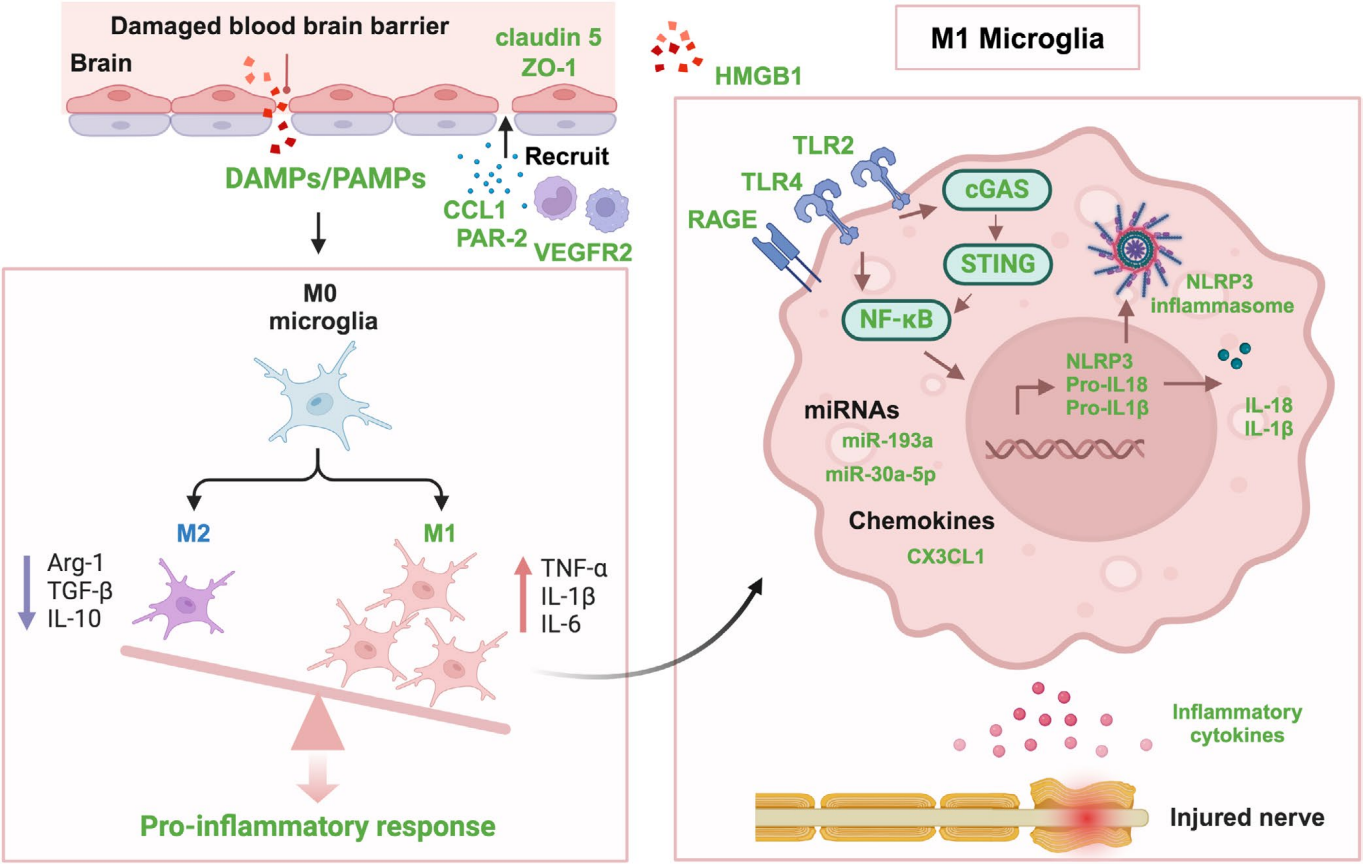
Microglia mediated neuroinflammation in post‐TBI cognitive impairment. The inflammatory response mediated by microglia includes the release of pro‐inflammatory cytokines, disruption of BBB, recruitment of immune cells, interference with neuronal function, and inhibition of nerve repair and regeneration. CCL1, chemokine C‐C motif ligand 1; DAMPs, damage associated molecular patterns; HMGB1, high mobility group box 1; NLRP3, NOD‐pyrin‐domain‐containing protein 3; PAR‐2, proteinase‐activated receptor‐2; PMAPs, pathogen‐associated molecular patterns; RAGE, receptor for advanced glycation end product; TLR, toll like receptor; VEGFR2, vascular endothelial growth factor receptor 2; ZO‐1, zonula occludens‐1.

In summary, the inflammatory response mediated by microglia participates in cognitive impairment after TBI through various pathways, including the release of pro‐inflammatory cytokines, induction of oxidative stress, disruption of the BBB, recruitment of immune cells, interference with neuronal function, and inhibition of nerve repair and regeneration (Figure 2).

### 2.3 Synaptic Plasticity Dysfunction

Synaptic plasticity‐related molecules are considered essential for maintaining normal brain function. More and more evidence suggested that TBI can induce long‐term memory deficits, which may be directly related to the activation of microglia in rodents, decreased synaptic plasticity, and abnormal expression of synaptic proteins [54]. Synaptic plasticity and biomarkers of learning and memory have also been applied to evaluate brain injury in TBI models. A study observed the temporal characteristics of axonal and somal injury that may lead to cognitive impairment in a mild TBI rat model. It was found that after mild lateral fluid percussion brain injury at 4, 24, 72 h, 4, and 6 weeks, there was little change in the level of neuronal cell body damage and myelin integrity, while axonal damage showed significant changes. At 4 h after injury, axonal damage was limited to the cingulate cortex, while at 4 and 6 weeks, significant axonal damage appeared on the outer capsule, and axonal damage appeared in the dorsal thalamic nucleus at 6 weeks. Three weeks after injury, the memory ability of TBI rats showed impairment, which may be related to sustained axonal damage [55].

**FIGURE 3:**
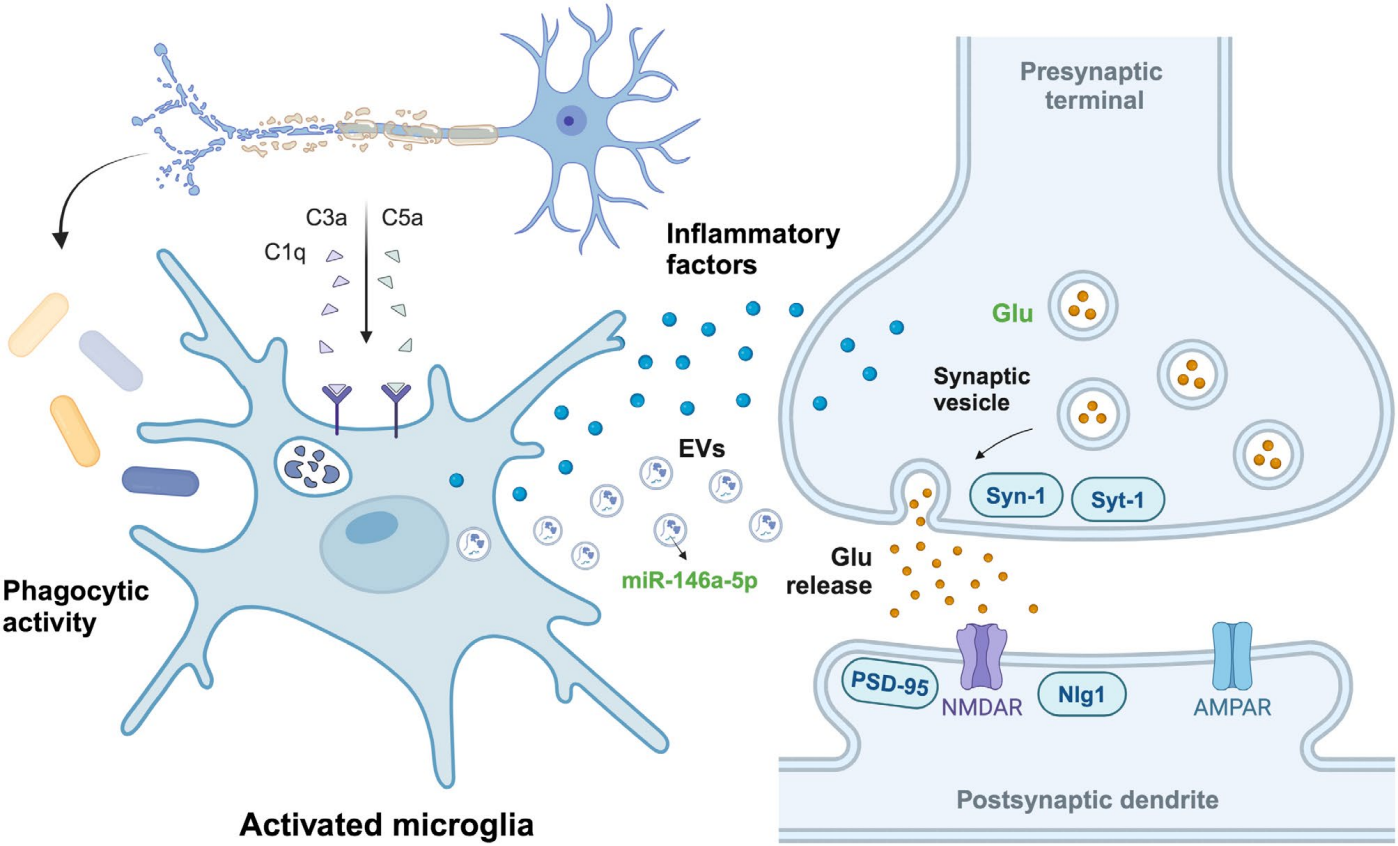
Microglia mediated synaptic plasticity in post‐TBI cognitive impairment. Complement activation can mediate the phagocytosis of microglia at synapses and impair the function of neural networks. Microglia can produce extracellular vesicles (EVs) carrying miRNA, regulate the expression of key synaptic proteins, and affect dendritic spine density in hippocampal neurons. EVs, Extracellular vesicles; Glu, glutamate; Nlg1, neuroligin1; NMDAR, N‐methyl‐D‐aspartate receptor; Syn1, synapsin‐1; Syt1, synaptotagmin.

First, microglia have phagocytic activity and can clear fragments of neurons and synapses. However, in the inflammatory environment after TBI, the phagocytic activity of microglia may be overactive, leading to excessive clearance of normal synapses and damaging the function of neural networks [56]. Research has shown that abnormal activation and phagocytic activity of microglia are closely related to synaptic loss and cognitive dysfunction in some neurodegenerative diseases [57]. A study utilized flow cytometry to quantify pre‐ (synapsin‐1) and post‐ (PSD‐95) excitatory synaptic markers. It was found that while an increase in phagocytic phenotype was observed in microglia, the number of excitatory synapses significantly decreased, regardless of the gender of the mice. After TBI, microglia are wrapped around neuronal cell bodies, known as the perineuronal “satellite microglia”. There were spatial interactions between microglia and dorsal hippocampal neurons. The synaptic contact between microglial cells and synaptic elements increases, and microglia are parallel to spinal cord and neuronal cells, replacing the presynaptic terminals of axons, which may be the reason why the phagocytic phenotype in microglia leads to synaptic loss [58]. Complement activation can mediate the microglial phagocytosis of synapses [59]. In a mouse model of TBI, the number and phagocytic activity of microglia increased 30 days after injury, and the synaptic density of hippocampal neurons decreased. Along with the significant accumulation of complement C1q, C3, and CR3, which can regulate the interaction between microglia and synapses in the hippocampus, further significant memory defects were produced [60]. In primary culture of microglia, treatment with the neurotransmitter glutamate can also increase the expression of synaptic phagocytic related genes such as CD11b, CX3CR1, and cathepsin S, thereby enhancing the phagocytic activity of microglia [61]. The enhanced synaptic phagocytosis of microglia can cause BBB damage, further leading to the invasion of T cells and other cells into the brain, resulting in more severe inflammatory reactions [62]. Secondly, the inflammatory mediators released by the activation of M1‐type microglia can affect the structure and function of synapses, as well as induce synaptic loss and neuronal degeneration, further disrupting the stability of the neural network. Studies have found that inflammatory microglia can produce extracellular vesicles (EVs) carrying miRNAs that can regulate the expression of key synaptic proteins, such as miR‐146a‐5p. It can inhibit the expression of pre‐synaptic synaptotagmin1 (Syt1) and post‐synaptic neuroligin1 (Nlg1), adhesion proteins that play a key role in dendritic spine formation and synaptic stability, leading to a decrease in dendritic spine density in hippocampal neurons [63] (Figure 3).

## 3 Therapeutic Approaches of Targeting Microglia‐Related Signaling in Post‐TBI Cognitive Impairment

Due to the lack of specific drugs that can prevent the progression of secondary injuries, the management of cases of post‐TBI cognitive impairment is mainly supportive at present. However, with the development of science and technology, the therapeutic approaches of targeting microglia‐related signaling have great potential and clinical application prospects in the management of post‐TBI cognitive impairment. The therapeutic methods targeting microglia mainly involve inhibiting their excessive activation, reducing inflammatory responses, and regulating the phenotypic transformation of microglia, etc. (Table 1).

**TABLE 1:** Therapeutic applications of glial cells in post‐TBI cognitive impairment.

Refs.	Model	Intervention methods	Parameters	Related behaviors	Test sites	Biochemical measurements
Zhang, 2022 [42]	Weight‐drop plus blood loss reinfusion	C‐176	Intraperitoneal injection once after modeling	NSS, NOR, OFT, FST	Hippocampus	TBK1↓, IRF3↓, IFN‐β↓, caspase‐1↓, GSDMD↓, NLRP3↓, M1‐MG↓
Zhang, 2021 [64]	CCI	IL‐4 nanoparticles	Repetitive intranasal, 4 weeks	NOR, passive avoidance test	Hippocampus	PPARγ↑, arginase‐1↑, LTP↑, M2‐MG↑
Okuma, 2019 [65]	FPI	Anti‐HMGB1 monoclonal antibodies	Intravenous injection, 14 days	Accelerated rotarod test, MWM	Cortex	CD68‐positive MG↓, HMGB1↓
Tentu, 2023 [66]	CCI	Oxyberberine, oxyberberine‐nanocrystals	3, 25, 50 mg/kg/day	OFT, MWM, NOR	Cortex	TLR4↓, HMGB1↓, NF‐κB↓, TNF‐α↓, IL‐6↓
Bachstetter, 2016 [67]	FPI	MW01‐2‐151WH (= MW151)	0.5–5.0 mg/kg, intraperitoneal injection	MWM	Cortex	IL‐1β↓
Huang, 2021 [68]	CCI	DP, Pom	0.5 mg/kg, 5 h post injury	OFT, NOR	Hippocampus	Microgliosis/Astrogliosis↓, neurons↑
Mao, 2011 [69]	Weight‐drop	PACAP	Intracerebroventricular pretreatment	MWM, mNSS, the inclined plane task	Cortical brain tissue, hippocampus	TLR4↓, MyD88↓, p‐IκB↓, NF‐κB↓, IL‐1β↓, TNF‐α↓
Liu, 2019 [70]	Weight‐drop	bpV(pic)	Intraventricular injection, 20 μg/100 g, 4 times	NSS, MWM	Brain tissue	AKT↑, IL‐1β↓, TNF‐α↓, CD32↓, Arg‐1↑, IL‐10↑, TGF‐β↑, NF‐κB p65↓
Shi, 2015 [71]	CCI	Ethyl pyruvate	Intraperitoneal injections, 30 mg/kg	Foot‐fault test, MWM	Brain, blood	IL‐1α↓, IL‐1β↓, IL‐6↓, TNF‐α↓, COX‐2↓, iNOS↓
Si, 2020 [47]	CCI	miR‐193a antagomir	0.5 nmol/day, 6 days	MWM	Hippocampus	CD68↓, CCL5↓, IL‐6↓, IL‐1β↓, TNF‐α↓, COX2↓, NLRP3↓, ASC↓, Caspase‐3↓
Zhang, 2020 [72]	CCI	miR‐711	Tail vein injection	mNSS, MWM	Brain, blood	M2/M1↑, Itpkb↓, TNF‐α↓, IL‐10↑
Hu, 2024 [48]	CCI	LV‐shLINC00707	Intraventricular injection	mNSS, MWM	Cortex, blood	IL‐6↓, TNF‐α↓
Chen, 2024 [73]	CCI	Neonatal microglia transplantation	2 μL	OFT, MWM	Cortex	NLRP3↓, M1/M2 ratio↓, TNF‐α↓, IL‐10↑
Brabazon, 2017 [74]	CCI	Insulin	Intranasal injection, 14 days	Beam walking assay, MWM, PET/CT with [^18^F]‐FDG imaging	Hippocampus	Hippocampal lesion volume↓, glucose uptake↓, microglia/macrophages↓
Gao, 2006 [75]	FPI	hNSCs transplantation	1 day post‐TBI	MWM	Hippocampus	GDNF↑, neurons↑
Sun, 2020 [76]	Weight‐drop	Curcumin	Intraperitoneal injection, 10, 20, 30, 50 mg/kg	MWM	Hippocampus	IL‐1β↓, IL‐6↓, IL‐18↓, TNF‐α↓, NLRP3↓, BDNF↑, p‐TrkB↑, PI3K↓, Akt↓
Moschonas, 2023 [77]	CCI	MBIs	3 h/day, 19:00 to 22:00 h, 30 days	Beam‐walk, cognitive (acquisition of spatial learning and memory), anxiety‐like behavior (open field), coping (shock probe defensive burying)	Hippocampus	BDNF↑, M1‐MG↓
Liu, 2017 [78]	Weight‐drop	Posttraumatic hypothermia pretreatment	Cooled twice (once a week), 2 weeks before TBI treatment	NSS, Hindlimb extension reflex, rotarod, OFT, elevated plus maze, NOR, MWM	Cortex and hippocampus	PSD93↑, PSD95↑, NR2B↑, spine number↑, LTP↑, M1‐MG↓
Laura, 2020 [79]	CCI	Physical exercise	3/7 weeks of exercise initiated 4 days post‐injury	NOR	Hilus, hippocampus	NeuN^+^ cells↑, DCX^+^ cells↑, M1‐MG↓
Kumar, 2014 [80]	Weight‐drop	*Panax ginseng* and minocycline	*Panax ginseng* (50, 100, 200 mg/kg) with minocycline (25, 50 mg/kg)	MWM, memory retrieval test	Cerebral cortex, hippocampus	TNF‐α↓, IL‐6↓, AChE↓, MDA↓, nitrite↓, GSH, SOD↑, catalase↑

Abbreviations: AChE, acetyl cholinesterase; CCI, controlled cortical impact injury; FPI, fluid percussion injury; Itpkb, 1,4,5‐trisphosphate 3‐kinase B; MBIs, music based interventions; MDA, malondialdehyde; mFPI, midline fluid percussion injury; mNSS, modified neurological severity score; MWM, Morris water maze test; NOR, novel object recognition test; NR2B, N‐methyl‐D‐aspartate receptor subtype 2B; NSS, neurological severity score; OFT, open field test; PSD93, postsynaptic density protein 93; PSD95, postsynaptic density protein 95; SOD, superoxide dismutase.

### 3.1 Anti‐Apoptosis

Under excitatory toxicity or oxidative stress, microglia are activated, releasing inflammatory mediators that overactivate Poly ADP ribose polymerase (PARP) in the neurons, deplete nicotinamide adenine dinucleotide (NAD^+^) (the main coenzyme in energy metabolism) and ATP, and lead to the release of apoptosis‐inducing factor (AIF) from mitochondria, inducing the apoptosis‐like pathway of PARP‐dependent cell death (parthanatos) [81]. A study has found that intranasal administration of NAD^+^ (20 mg/kg) can downregulate the number of activated microglia, protecting neurons in hippocampal CA1, CA3, and dentate gyrus of TBI rats, but not cortical neurons [82]. HET0016 is a specific inhibitor of 20‐hydroxyeicosatetraenoic acid synthesis, which can reduce the volume of brain damage after TBI and inhibit neuronal pyroptosis by downregulating the expression of NLRP3, caspase‐1, and gasdermin D (GSDMD) proteins. After TBI, inflammation and cell death can lead to the loss of a large number of nerve cells, resulting in neurological dysfunction. At the same time, the expression of p‐p38 MAPK and NF‐κB p65 in neurons and microglia was also significantly reduced, alleviating the neuroinflammatory response after TBI [83].

### 3.2 Anti‐Inflammation

The chronic inflammatory response induced by inflammatory glial cells can lead to neurodegeneration and loss. A study conducted immunohistochemical detection of reactive microglia (CD68 and CR3/43) in human autopsy brain tissue, and the main finding was that neuroinflammatory reactions appeared, developed, and persisted for several months within the first week after TBI [84]. Inhibiting the glial cell‐related inflammatory pathway may be one of the targets for improving cognitive impairment after TBI. Studies have found that inhibition of STING can reduce the number of activated glial cells, such as A1‐type astrocytes and M1‐type microglia, and restore neuronal integrity in the CA1 region, improving cognitive impairment in severe TBI rats [42]. IL‐4 is an anti‐inflammatory cytokine that can polarize microglia toward an anti‐inflammatory phenotype via receptor binding [85]. After 4 weeks of repetitive intranasal delivery of IL‐4 nanoparticles, it can increase the expression of PPARγ and arginase‐1 in microglia, promote the transformation of microglia to an anti‐inflammatory phenotype and improve hippocampal‐dependent spatial and non‐spatial cognitive function in TBI mice [64]. The damage caused by TBI directly leads to cell damage and death, and HMGB1 in the nucleus is passively released into extracellular mechanisms. At the same time, some activated immune cells also secrete HMGB1. The anti‐HMGB1 monoclonal antibody significantly inhibited HMGB1 translocation and reduced the accumulation of activated microglia in the cortex of the ipsilateral hemisphere. It also prevented neuronal death in the ipsilateral hippocampus after TBI and had a positive impact on EEG activity. The beneficial effects of anti‐HMGB1 monoclonal antibodies on motor and cognitive function can last up to 14 days after injury [65]. Natural products, such as berberine, also exhibit excellent neuroprotective effects. Some researchers synthesized oxyberberine (OBB) from berberine and prepared OBB‐nanocrystals (OBB‐NC). They further found that OBB or OBB‐NC administration can reduce long‐term neuropsychiatric complications such as anxiety, depression, and cognitive impairment, as well as inhibit the HMGB1‐mediated TLR4/NF‐κB pathway in microglia by downregulating the levels of TLR4, HMGB1, NF‐κB, TNF‐α and IL‐6 [66]. Other natural products, like curcumin, can also reduce hippocampal neuroinflammation by inhibiting activated microglia and declining inflammatory factors in TBI rats, which may be related to BDNF/Trkb/PI3K/Akt signaling [76].

MW151 (MW01‐2‐151WH) is a small molecule experimental therapeutic drug for central nervous system permeability, which can inhibit the excessive production of pro‐inflammatory cytokines caused by TBI. In a mild TBI model, it was found that MW151 administration can inhibit acute cytokine upregulation (such as IL‐1β), protect glial cell responses (microglia and astrocytes), and alleviate downstream cognitive impairment [67]. A kind of immunomodulatory imide drug (IMiD), named 3,6′‐dithiopomalidomide (DP) and Pom, can reduce the number of degenerated neurons in the hippocampal CA1 and dentate gyrus (DG) regions and damage brain volume after TBI. DP (rather than Pom) can significantly reduce TBI‐induced proliferation of microglia and astrocytes, and improve short‐term memory deficits and anxiety‐like behavior [68]. The pituitary adenylate cyclase‐activating polypeptide (PACAP), which belongs to the secretin/glucagon/vasoactive intestinal peptide (VIP) superfamily, has immunomodulatory properties [86]. PACAP pretreatment can inhibit the expression of TLR4 and its downstream signaling molecules MyD88, p‐IκB, and NF‐κB in microglia and neurons, while suppressing the levels of inflammatory factors such as IL‐1β and TNF‐α, reducing neuronal apoptosis and brain edema, and significantly improving motor and cognitive dysfunction of a weight‐drop model of TBI [69]. PTEN, called phosphatase and tensin homolog deleted on chromosome 10, is a tumor suppressor that plays an important role in mediating intracellular signaling pathways for cell proliferation and survival. Bisperoxovanadium (pyridine‐2‐carboxyl) [bpV(pic)] is an inhibitor of PTEN, which can activate the AKT pathway to inhibit PTEN lipid phosphatase activity, thereby exhibiting neuroprotective effects [87]. Research has found that injecting bpV (pic) can activate AKT and inhibit NF‐κB p65 expression, promote the transformation of microglia to M2 anti‐inflammatory phenotype, alleviate neuroinflammation, and significantly reduce brain edema and neurological dysfunction after TBI. Considering MCP‐1 has the ability to recruit monocytes and macrophages to promote inflammation, studies have found that BpV (pic) therapy can also inhibit the expression of MCP‐1 through the AKT/NF‐κB p65 signaling pathway [70]. After TBI, the structure and function of the BBB are disrupted, and the activation of resident glial cells and the invasion of peripheral immune active cells through the damaged BBB exacerbate neuroinflammatory responses [4]. Ethyl pyruvate (EP) is a stable lipophilic ester derivative of pyruvic acid, with strong free radical scavenging, anti‐inflammatory, and anti‐apoptotic properties [88]. It is reported that EP inhibits the activation of microglia and the release of inflammatory mediators, as well as the production of matrix metalloproteinase (MMP)‐9 by peripheral neutrophils, reducing the number of neutrophils with excessive MMP‐9 production in the spleen, thereby alleviating MMP‐9 mediated BBB rupture and brain edema. EP improved sensory motor and cognitive function 28 days after TBI [71]. Laquinimo is an oral neuroimmune modulator that can inhibit the infiltration of monocytes into the brain, and hierarchical clustering showed that the gene expression of microglia in the TBI group treated with Laquimod was more similar to that in the sham group than in the TBI control group [89].

Regulating the expression of miRNAs is also an important intervention method for treating cognitive impairment after TBI. The miR‐193a hairpin inhibitor (antagomir) can significantly reduce the damage volume, brain water content, and neuronal death in TBI mice induced by CCI. It can inhibit microglial activation, reduce the expression of neuroinflammatory markers, including CCL5, IL‐6, IL‐1β, TNF‐α, COX2, and significantly improve cognitive impairment. Further investigation revealed that it may be related to the blockade of the NLRP3 inflammasome [47]. It was found that miR‐124‐3p in microglial exosomes can promote the polarization of microglia toward anti‐inflammatory M2 and inhibit neuroinflammatory responses. Microglial exosomal miR‐124‐3p can also promote the growth of neural processes after scratch injury, including an increase in the number of neural process branches and total neural process length, as well as a decrease in the expression of RhoA and neurodegenerative proteins (Aβ‐peptide and p‐Tau), which may be related to the inhibition of the mTOR signaling pathway [90]. Microglia‐derived EVs carrying miR‐711 can target and inhibit 1,4,5‐trisphosphate 3‐kinase B (Itpkb), thereby inhibiting p‐Tau and increasing the M2/M1 ratio, reducing the neurological deficit score of TBI mice, and improving cognitive function [72]. MiR‐193a, miR‐124‐3p, miR‐711, and others may be promising therapeutic targets for treating TBI‐related neuroinflammation. Except regulating miRNAs, a study found that inhibiting LINC00707 can suppress the activation of microglia and excessive production of inflammatory factors, thereby improving brain edema and cognitive impairment in TBI rats, which may be achieved by targeting miR‐30a‐5p [48].

The recent views suggest that adult microglia mainly produce inflammation after injury, while the involvement of neonatal microglia after injury may be related to inflammation inhibition. This suggests that using neonatal microglia to alleviate neuroinflammation may be a promising approach for treating cognitive impairment [91]. It was found that transplantation of neonatal microglia significantly reduced anxiety‐like behavior and improved cognitive impairment in TBI mice. This may be achieved by reducing the levels of NLRP3, M1/M2 ratio, and inflammatory cytokine (TNF‐α), while increasing the level of anti‐inflammatory factor IL‐10 [73]. In addition, regulating brain energy metabolism can indirectly alleviate neuroinflammatory reactions caused by microglia. A study found that intranasal insulin can increase glucose uptake capacity after brain injury, reduce hippocampal lesion volume, and decrease microglia/macrophages in the hippocampus, but not astrocytes, thereby alleviating the inflammatory response and improving memory and learning function [74].

### 3.3 Regulating the Structure and Function of Synapses

To prevent the progression of long‐term damage caused by TBI, regulating synaptic plasticity is also crucial. In cases with diffuse traumatic axonal injury, the microglial activation is particularly pronounced in the white matter [84]. After TBI, complement activation persists in the injured brain, triggering a strong chronic neuroinflammatory response that extends to both hemispheres, mediating the phagocytosis of synapses by microglia. When complement activation is inhibited, it can interrupt the degenerative neuroinflammatory response and reverse cognitive decline. At the same time, research has found that treatment may be suitable at all stages of TBI, including acute and chronic time points after TBI [59]. Laquimod can not only inhibit the inflammatory response mediated by microglia in the TBI model, but also repair axonal injury and restore neurogenesis [89]. Repetitive intranasal delivery of IL‐4 nanoparticles can also improve hippocampal long‐term potentiation (LTP) function and repair hippocampal structure, including CA3 neuron loss, diffusion tensor imaging of white matter tracts, thereby enhancing cognitive ability [64]. Natural products, such as curcumin, can enhance neurogenesis by upregulating DCX (a marker of immature neurons at an early stage of neurogenesis) in the hippocampus and improve hippocampal‐dependent spatial memory of TBI rats [76].

### 3.4 Other Therapies

Although the above medication therapies have brought benefits in the laboratory, they have not yet been successfully translated into clinical practice. Traditional medicines have great prospects due to their advantages of minimal side effects, high safety, and low cost. Compared with single treatment, studies have found that the combination of *Panax ginseng* and minocycline (microglial inhibitor) can alleviate oxidative stress, neuroinflammation, and AChE levels in the cerebral cortex and hippocampus of traumatized rats; it can also alleviate cognitive impairment [80]. In addition to medication therapy, considering the personal, medical, and social burden of TBI patients, some complementary and alternative therapies have good therapeutic effects, such as music‐based interventions (MBIs), hypothermia pretreatment, and physical exercise. It was found that 30 days of classical music can reduce cortical injury volume and activate microglia, increase BDNF expression in the hippocampus, and thus improve motor, cognitive, and anxiety‐like behavior in TBI rats [77]. Hypothermia has been proven to reduce brain metabolism and oxygen consumption, alleviate edema, repair the BBB, improve survival rate and prognosis in TBI animal models, and has also been widely used in clinical practice [92]. It is reported that posttraumatic hypothermia can significantly inhibit microglia activation, increase the expression of PSD‐93, PSD‐95, and NR2B in the cortex and hippocampus, and restore the reduction in spine number and LTP damage as well as glucose metabolism levels 1 month after TBI, thereby attenuating cognitive deficits [78]. Research has found that physical exercise can also alleviate cognitive decline associated with TBI. Running can increase the number of neuroprotective cells (NeuN^+^) and neurogenic cells (DCX^+^) in the hilus and inhibit the activation of microglia in the dorsal hippocampus. It was also found that whether starting shortly after injury or after a period of time, exercise has benefits for improving memory [79].

Stem cell therapy is currently a promising treatment method in TBI [93]. Stem cells are characterized by self‐renewal and multipotent differentiation potential and can replace lost neurons and glial cells in TBI [94]. Serdar Kabatas et al. conducted a single center, prospective, longitudinal medical trial aimed at evaluating the safety and efficacy of Wharton's Jelly‐derived mesenchymal stem cells (WJ‐MSCs) in treating TBI. It was found that after stem cell transplantation, TBI patients only experienced early and transient complications such as hypothermia, mild headache, and muscle pain, which disappeared within 24 h. During a one‐year follow‐up, they showed improvements in cognitive ability, muscle spasms, muscle strength, performance scores, and fine motor skills [95]. A study transplanted primed human fetal neural stem cells (hNSCs) into the brains of TBI rats and found that after receiving hNSCs transplantation for 10 days, hNSCs survived and mainly differentiated into neurons in the damaged hippocampus, releasing glial cell‐derived neurotrophic factor (GDNF) and improving spatial learning and memory abilities in rats [75]. It is found that intranasal injection of human MSC‐derived extracellular vesicles can prevent chronic brain dysfunction after TBI by inhibiting NLRP3‐p38/MAPK signaling in activated microglia [96]. Researchers have transplanted human umbilical cord‐derived mesenchymal stem cells (hUC‐MSCs)‐derived mitochondria into a TBI rat model and found that the successful internalization of mitochondria in neuronal cells significantly reduced the number of apoptotic brain cells, reduced microglial activation, maintained normal brain morphology and cell structure, and improved symptoms in TBI rats [97].

Some other adjuvant therapies, such as acupuncture, have also been shown to have good prospects in improving cognitive impairment after TBI [98]. It is reported that electroacupuncture (EA) can inhibit the activation of microglia, reduce the levels of inflammatory factors, and alleviate further damage to brain tissue caused by neuroinflammation [99]. It can also promote the survival and synaptic remodeling of damaged neurons by regulating the expression of brain‐derived neurotrophic factors and glial cell‐derived neurotrophic factors [100]. In addition, combination therapy has also been widely used in the treatment of post‐TBI cognitive impairment. Acupuncture combined with cognitive training, hyperbaric oxygen, physical factors, as well as traditional or Western medicine also has a potential effect on improving cognitive impairment after TBI [101].

## 4 Discussion and Conclusions

TBI is the strongest environmental risk factor for the development of AD and other dementia‐related neurodegenerative diseases. The cognitive impairment caused by TBI often involves extensive damage and death of neurons. The limited regenerative capacity of neurons in the central nervous system makes it extremely challenging to restore normal cognitive function after repairing damage.

The acute phase of TBI refers to the period from a few hours to several days following the injury. Whether it is the damage caused directly by mechanical force (such as neuronal death, axonal destruction, and vascular rupture) or secondary brain injury exacerbated by oxidative stress or acute inflammatory response. Microglia play a complex and critical role in cognitive impairment after TBI, with a “double‐edged sword” effect. Their dynamic phenotype transition and spatiotemporal specificity often determine neural outcomes. Within minutes after injury, microglia sense DAMPs through surface receptors such as TLR4 and complement receptors, rapidly activating and transforming into amoeboid‐like morphology. They secrete pro‐inflammatory cytokines and chemokines to recruit peripheral immune cells to the site of injury. Microglia polarization is mainly characterized by M1 pro‐inflammatory phenotype, with high expression of markers such as iNOS and CD86, releasing ROS and NO, and exacerbating neuronal damage through pathways such as TLR4/NF‐κB [102]. The chronic phase of TBI often lasts for weeks to years, and the persistent presence of chronic inflammation is the main characteristic, which is associated with an increased risk of neurodegenerative diseases and accompanied by long‐term neurological and psychiatric sequelae such as cognitive impairment. The cognitive impairment discussed in this review often occurs during this stage. As cells with long lifespan and low renewal rate, microglia are easily affected by TBI in the long term. From weeks to months, microglia enter a prime state, characterized by upregulation of antigen‐presenting molecules and reduced cytokine secretion. At the same time, increased responsiveness to secondary stimuli (such as infection and stress) can easily lead to excessive inflammation. Trauma‐associated microglia continue to activate after brain injury, and studies have found that IFN‐related genes (Ifitm3, Isg15, Ifi27l2a, Irf7, and Stat1) and messenger activation‐related genes (Trem2, Apoe, Clec7a, H2‐D1, and H2‐K1) that mediate inflammatory responses can be detected [103]. From months to years, trauma‐associated microglia continue to activate and exhibit a mixed phenotype (simultaneously expressing M1/M2 markers), which promotes synaptic pruning abnormalities and neuronal loss, leading to cognitive impairment [104]. The acute and chronic staging of TBI reflects the dynamic process of inflammation and repair balance. In the acute phase, microglia exacerbate secondary damage through M1 polarization, while in the chronic phase, long‐term cognitive dysfunction is caused by activation and inflammatory response. Most current treatment methods can only alleviate symptoms and are difficult to promote complete repair of neurons. Future research may need to combine single‐cell sequencing and metabolomics to analyze heterogeneous subpopulations of microglia at different time points and develop time‐specific treatment strategies.

The role of microglia in the inflammatory response and nerve damage after TBI has been widely studied. Previous reviews have mainly focused on the phenotype transition of microglia M1/M2 and the release of inflammatory factors, but there is a lack of systematic exploration of the dynamic changes in phagocytic function and its direct association with cognitive impairment. In this review, the “double‐edged sword” effect of microglial phagocytic function is proposed, and the spatiotemporal dynamic changes of microglial phagocytic function after TBI are systematically summarized. At the same time, the latest research supplemented the explanation of how complement‐mediated synaptic pruning mechanism leads to hippocampal‐dependent memory impairment and supplemented the roles of various miRNAs. The system has organized the mechanisms of cognitive impairment mediated by microglia after TBI, including apoptosis, neuroinflammation, synaptic plasticity, etc., and systematically discussed in the review the remote regulatory effects of inflammatory factors and non‐coding RNA carried by microglia EVs on neuronal synapses.

Targeted regulation of microglial activation status in treatment methods will help reduce chronic inflammatory reactions and improve cognitive function. Previous treatment reviews have focused on anti‐inflammatory drugs and phenotype switching regulation, lacking multi‐target combination strategies and integration of new technologies. We are concerned about integrated traditional Chinese and Western medicine strategies such as acupuncture, stem cell therapy, and music therapy that can provide new hope for promoting neuronal regeneration and nerve repair. The mechanism of stem cell therapy for post‐TBI cognitive impairment covers multiple dimensions such as nerve regeneration and inflammation regulation, etc. Novel strategies based on WJ MSCs, extracellular vesicles, and genetic engineering are proposed to specifically regulate the polarization phenotype of microglia, promote the secretion of anti‐inflammatory factors by microglia, and inhibit the expression of pro‐inflammatory genes, which are expected to break through the existing treatment bottleneck. The paracrine effect mediated by extracellular vesicles further enhances the safety of stem cell therapy. Although it still faces challenges such as standardized preparation and optimal transplantation protocols, existing clinical data have confirmed its safety and effectiveness, and the current clinical research has also moved from safety verification to precise regulation. Acupuncture and music therapy, with the advantages of multi‐target regulation, low risk, and easy acceptance, have gradually become an important part of the treatment strategy for cognitive impairment after TBI. These therapies are demonstrating potential application value in preclinical studies. Strengthening basic research and large‐scale clinical trials to develop more effective treatment plans targeting microglia will bring better quality of life and rehabilitation effects to patients with cognitive impairment after TBI.

In conclusion, this review focuses on the key role of microglia in post‐TBI cognitive impairment and provides a detailed introduction to potential treatment methods. This includes inhibiting the activation of microglia and the release of inflammatory factors through anti‐inflammatory therapy, regulating the phenotype of microglia to promote their transformation to M2 type, promoting the normalization of microglial phagocytic function, regulating the structure and function of synapses, and using stem cell therapy to secrete neurotrophic factors to regulate microglial function. Through in‐depth research on the pathological changes and treatment methods of microglia in post‐TBI cognitive impairment, it is expected to provide an important theoretical basis and treatment strategies for improving cognitive function in patients with TBI.

## Author Contributions

Conceptualization: L.Z., X.H., and N.L.; methodology: N.L., W.L., and L.T.; software: L.Z., H.L., and C.H.; data curation: J.J., J.Z., and S.C.; writing – original draft: L.Z., X.H., and N.L.; writing – review: L.Z., X.H., and N.L.; writing – editing, W.L., L.T., L.Z., and H.L.; supervision: C.H., J.J., J.Z., and S.C.; funding acquisition: L.Z., X.H., and N.L. All authors read and approved the final manuscript. All authors contributed to data collection, analysis, drafting, and revising the article, gave final approval of the version to be published, and agreed to the submitted journal.

## Ethics Statement

The authors have nothing to report.

## Consent

The authors have nothing to report.

## Conflicts of Interest

The authors declare no conflicts of interest.

## Data Availability

The data that support the findings of this study are available from the corresponding author upon reasonable request.
